# Practical long‐term storage of strawberries in refrigerated containers at ice temperature

**DOI:** 10.1002/fsn3.1817

**Published:** 2020-08-05

**Authors:** Atsushi Ikegaya, Seiji Ohba, Teruko Nakajima, Tomoyasu Toyoizumi, Seiko Ito, Eiko Arai

**Affiliations:** ^1^ Shizuoka Professional University Junior College of Agriculture Iwata Japan; ^2^ Shizuoka Prefectural Research Institute of Agriculture and Forestry Iwata Japan; ^3^ Graduate School of Integrated Pharmaceutical and Nutritional Sciences University of Shizuoka Shizuoka Japan; ^4^ School of Food and Nutritional Sciences University of Shizuoka Shizuoka Japan

**Keywords:** organic acid, sensory evaluation, storage temperature, strawberry, sugar

## Abstract

This study investigated the effect of storage temperature in the presence or absence of film packaging on the Benihoppe and Kirapika varieties of Japanese strawberries stored for 28 days at 0°C and 3°C. The study was conducted in a 20‐foot reefer container for practicality. Storage at 0°C suppressed decay and reduction in sugars and organic acids more efficiently than that at 3°C. Softening of fruit hardness was also suppressed depending on the variety. The reduction in sugars and organic acids did not affect strawberry palatability. Along with low temperature, long‐term storage of strawberries also requires the use of film packaging, which prevents drying. Without film packaging, storage at both 0°C and 3°C decreased fresh weight significantly, resulting in loss of commercial value. In contrast, storage in film packaging decreased weight reduction to <5%, even after 28 days cold storage.

## Graphical abstract

Storage at 0°C suppressed decay, and reduction of sugars and organic acids more efficiently than that at 3°C. Along with low temperature, long‐term storage of strawberries also requires the use of film packaging, which prevents drying. Without film packaging, storage at both 0°C and 3°C decreased fresh weight significantly, resulting in loss of commercial value. The proposed method can enhance the storage period of strawberries to 28 days or more.

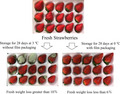

## 1 INTRODUCTION

The strawberry (*Fragaria × ananassa* Duchesne) is one of the world's most popular fruits. Strawberries are produced in many countries; however, their cultivars, cultivation methods, distribution systems, and consumption trends vary greatly with country (Angelini, 2010). Strawberries have an extremely thin fruit skin and soft flesh; thus, their shelf‐life is reported to be around 7 days, even when stored at a typical storage temperature of about 5°C (Ayala‐Zavala, Wang, Wang, & González‐Aguilar, 2004). Accordingly, a large number of studies have been conducted on strawberry storage and distribution technology development worldwide (Filho et al., 2018; Ktenioudaki, O’Donnell, & Nunes, 2019). Many reports indicate 0°C as the optimal storage temperature for strawberries because this temperature does not cause freezing and minimizes the occurrence of decay (Ayala‐Zavala et al., 2004; Park, Kim, & Hwang, 2012). Low temperature inhibits growth of the fungus *Botrytis cinerea*, which plays a significant role in the decay of strawberries (Ayala‐Zavala et al., 2004; Sallato, Torres, Zoffoli, & Latorre, 2007). However, in practice, it is difficult to accurately set refrigerators to 0°C. The temperature at various locations in a typical industrial refrigerator can differ by 2°C or more (Laguerre, Derens, & Palagos, 2002). Similar findings have been reported for the refrigerated containers used in transport, depending on the position in the refrigerator and the operating conditions of the defrost cycle (Rodríguez‐Bermejo, Barreiro, Robla, & Ruiz‐García, 2007). The freezing point of strawberries differs depending on their variety and ripeness, but it is generally reported to be around −1.1°C (Whiteman, 1977). Thus, the temperature at some of these locations may be lower than the freezing point of the strawberry. Freezing can destroy cell membranes and cause significant loss of storability (Morris & Clarke, 1981). Therefore, the recommendations of previous studies to store strawberries at 0°C have not been put to practical use, and a storage temperature of approximately 3°C has been adopted as a practical storage temperature.

However, new refrigerated containers that can maintain their set temperature more accurately have been developed in recent years. We anticipate that these could also be used to store strawberries at temperatures very close to 0°C, on a commercial scale and cost. Therefore, in this study, we used this practical refrigerated container rather than an incubator to conduct long‐term storage testing of strawberries at 0°C. Furthermore, the humidity and gas environment have a significant effect when storing fruits. As this container has no mechanism to control these factors, we considered using a film package to control them.

Th present studies on long‐term cold storage of fresh strawberries have been performed for 14–21 days (Ayala‐Zavala et al., 2004; Matsumoto et al., 2008). Thus, we aimed to examine storage for 28 days and beyond.

In addition, previous studies on strawberry storage have examined the appearance of decay and changes in the components of strawberries due to storage (Bang, Lim, Yi, Lee, & Lee, 2019; Pott et al., 2020; Yan et al., 2019). However, changes in the taste of strawberries due to storage have not been studied. Therefore, we evaluated the appearance, hardness, and natural compounds before and after storage, along with the taste. We investigated sugars and organic acids, the compounds that are abundant in strawberries and have a large effect on their taste (Rosenfeld & Ness, 2000).

## 2 MATERIALS AND METHODS

### 2.1 Plant materials

Fresh strawberries (Benihoppe and Kirapika varieties) were used for the storage test and sensory evaluation. Both varieties were grown using elevated cultivation in a test greenhouse at the Shizuoka Prefectural Research Institute of Agriculture and Forestry (Iwata, Shizuoka, Japan). The Benihoppe variety was originally developed by the same government agency and is grown in various areas of Japan (Takeuchi, Fujinami, Kawata, & Matsumura, 1999). Kirapika is a new variety, also developed by the same agency, as a successor to Benihoppe (Kawata et al., 2016). When harvesting strawberries for the test, all ripe fruits were harvested in advance on the previous day, and only those that had just ripened on the next day were used.

### 2.2 Strawberry packaging and storage

Each of the two strawberry varieties was tested with and without film packaging, at 0°C and 3°C. The ripe strawberries of each variety were harvested on 29 November 2016. For each variety, 15 strawberries were placed in a 15‐hole pack to achieve a weight of around 250 g.

For each variety, one pack was covered with freshness preserving film (P‐Plus, 0.03 × 380 × 270 mm, Sumitomo Bakelite Co., Ltd.), which was then heat sealed. The packaged and nonpackaged samples of each variety were then placed in the same cardboard shipping box (264 × 362 × 366 mm).

The shipping boxes containing the strawberry packs were precooled for 18 hr in a 3°C refrigerator and were then transferred to one of two precise temperature‐controlled refrigerated containers (Fresh Keeping Device “futecc,” Denso Corporation), one with the temperature set to 0°C, and the other set to 3°C and were stored for 28 days. For comparison, some of the precooled samples were not transferred to the refrigerated container (nonstored). The temperature of 0°C was chosen to represent the lowest actual temperature for storing strawberries without freezing, and 3°C was chosen to represent the lowest temperature at which they can be stored without freezing in a conventional reefer container. After 28 days of storage, the shelf life of the strawberries was evaluated by sealing them in a thermostat chamber for 24 hr at 20°C (Ozkaya, Dündar, Scovazzo, & Volpe, 2009), because abnormalities may occur with some fruits and vegetables after transferring from a low temperature to a normal temperature (Eaks & Morris, 1956).

The oxygen (O_2_) and carbon dioxide (CO_2_) levels inside the film were also measured using a headspace gas analyzer for packaging (CheckPoint II, Mocon Europe A/S) at the end of cold storage for 28 days.

### 2.3 Temperature and humidity during storage, and strawberry weight

A temperature and humidity logger (K‐295F, Fujita Electric Works Ltd.) were set up both inside the shipping box and inside the film packaging. Temperature and humidity were recorded every 10 min, starting immediately after the storage period began.

### 2.4 Fresh weight and decay

For fresh weight, the strawberry samples were first weighed before storage. Then, the strawberries were stored for 28 days, left at 20°C for 24 hr, and measured again. The fresh weight before storage was defined as 100%. Fungal decay was determined visually during the course of the experiment. Strawberries showing mycelial development on 5% or more of their surface were considered as decayed (Ayala‐Zavala et al., 2004). The number of decayed samples was counted among 30 samples in each treatment group.

### 2.5 Fruit hardness and color

To assess fruit hardness, samples of strawberries were removed from storage and left for 24 hr at 20°C in their packs. The fruit hardness of each individual strawberry was measured using a universal testing machine (RE2‐33005s, Yamaden Co., Ltd.). Each strawberry was divided into two parts from the peduncle to the fruit apex, placed on a sampling stage with the cut side downward, and then penetrated with a cylindrical plunger (No.4) with a diameter of 3 mm at the highest point on the strawberry, at a rate of 10 mm/s until the distortion factor reached 50%. The initial breaking stress in the obtained profile curve was set as the fruit hardness (Mochizuki et al., 2018).

The color of the fruit skin at the center of the fruit surface was measured using a colorimeter (CR‐221, Minolta, Inc.), and the colors were expressed using the L*a*b* color system formulated by the International Commission on Illumination (CIE 1976, CIELAB).

### 2.6 TSS, free sugar, and organic acid in the juice

To produce the juice, the strawberry calyxes were removed, and they were then individually wrapped in a 100‐mesh gauze and juiced in a juicer (Hand Juicer, Ito Seisakusho Co. Ltd.). The total soluble solid (TSS) content in the resulting juice was measured using a sugar refractometer (PAL‐1, Atago Co. Ltd.). The juice was subjected to refrigerated centrifugation for 10 min at 6,400 × *g* at 4°C. The supernatant was collected, diluted 50‐fold in water, and then filtered through a 0.45 µm filter. The levels of glucose, fructose, and sucrose, which are the main free sugars in strawberries (Paparozzi et al., 2018), were determined in the supernatant using high‐performance liquid chromatography (HPLC). The analysis conditions were as follows: HPLC, Alliance2695 (Waters); column, SC1011 (8 mm I.D. × 300 mm, Showa Denko K.K.); eluent, water; flow rate, 1 ml/min; detector, differential refractometer (2414, Waters); standard: glucose, fructose, and sucrose (Wako Pure Chemical Industries).

The same juice supernatant was diluted fivefold in water, filtered, and used for determining the levels of citric acid and malic acid, which are the main organic acids in strawberries (Koyuncu & Dilmaçünal, 2010), along with succinic acid using HPLC. The analysis conditions were as follows. HPLC, LC‐10AD‐Vp (Shimadzu Corporation); column: Shim‐pack SCR102H (8 mm I.D. × 300 mm, Shimadzu); eluent: 5 mM p‐toluenesulfonic acid aqueous solution; flow rate: 0.8 ml/min; detector: conductivity detector (CDD‐6A, Shimadzu); standard: citric acid, malic acid, and succinic acid (Wako Pure Chemical Industries).

Based on the presumption that all fruit weight reduction would be due to a reduction in water content, the values of the obtained TSS, total free sugar, and total organic acid were divided by the value 100 (%)—weight reduction portion (%), to control for the effect of compounds concentration by moisture depletion.

### 2.7 Sensory evaluation of juices before and after storage

The effect of storage on the taste of strawberries was also evaluated. Ripe strawberries of each variety were harvested on 9 April 2018. Half of the harvested lot were immediately juiced and frozen, and the other half were film packaged as described in Section 2.2, stored for 28 days at 0°C, and subsequently juiced and frozen (prestorage). Frozen samples were stored at −30°C. The juice samples of each treatment group were mixed before freezing. The taste of the juice samples from stored and fresh fruits was compared.

### 2.8 Analysis of juice used for sensory evaluation

Strawberry juices were analyzed for TSS, free sugar, and organic acid by the same method described in Section 2.6, prior to their sensory evaluations. In addition, the number of aerobic bacteria and fungi in strawberry juice was counted. Thawed strawberry juices were diluted 10 times with sterile water to reach 10^6^. For aerobic bacteria, diluted fruit juices were placed in a culture medium (Petrifilm for aerobic count, 3M Company), cultured at 35°C for 48 hr, and the colonies were counted. For fungi, diluted fruit juices were placed in a culture medium (Petrifilm for yeast and mold count, 3M Company) and cultured at 25°C for 72 hr, and the colonies were counted. The measurement was performed three times for each sample, and the average value was used. Microbial tests were performed according to the Association of Official Analytical Chemists (AOAC) official method.

### 2.9 Sensory evaluation

The stored juices were thawed at 20°C immediately prior to sensory evaluation. The taste of the juices was assessed by a panel of 40 participants from various age groups (7, 11, 9, 10, and 3 people in their 20s, 30s, 40s, 50s, and 60s, respectively), of both genders (21 females and 19 males), and with varying educational backgrounds. The juices were presented in plastic cups (30 ml) and coded with 3 random digits.

The attributes compared by the panelists were sweetness, sourness, flavor, and overall preference. The panelists assessed each attribute in a two‐sample difference test (Meilgaard, Civille, & Carr, 2007), choosing the sample that they felt to more fully match a particular attribute.

### 2.10 Statistical analysis

All measurements were performed at least in triplicate, and each value was expressed as the mean ± standard deviation (*SD*). Results were evaluated using ANOVA, and means were compared using Tukey's multiple range test for significance at a *p* value < .05. Angle conversion was applied in advance for the fresh weight test, thereby canceling out heteroscedasticity. In addition, the results of sensory evaluation were evaluated using a two‐tailed test for a significance level of 5%.

## 3 RESULTS

### 3.1 Temperature, humidity, and gas concentration during storage

**Figure 1:**
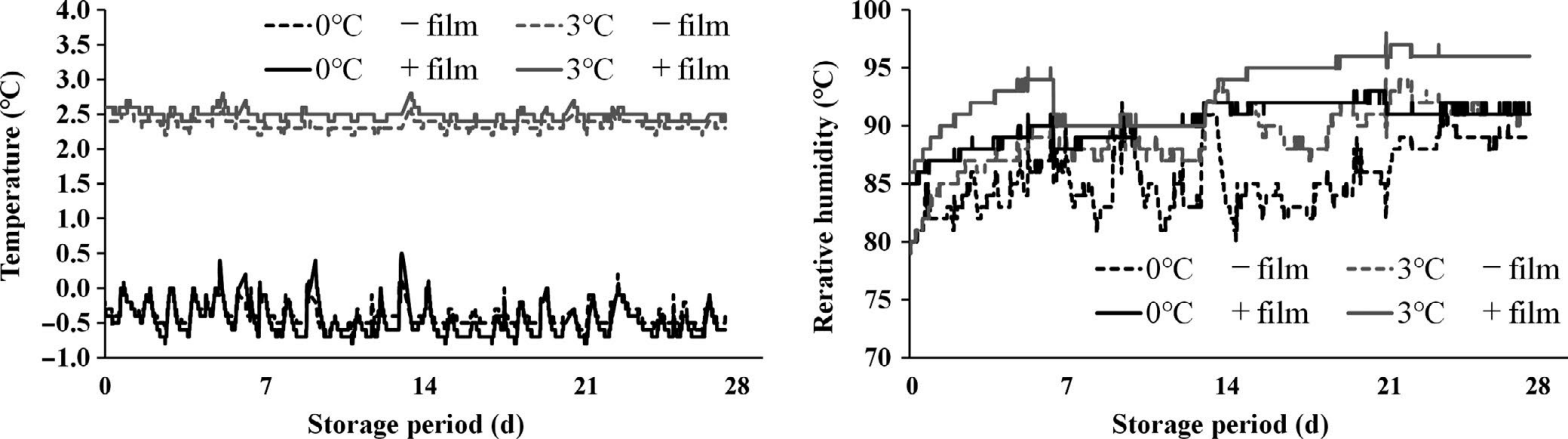
Temperature and humidity around the strawberries and inside the film packaging during the storage period. We recorded the temperature and humidity in the environment surrounding the “Benihoppe” strawberries stored under refrigeration at 0°C and 3°C. Strawberries without film packaging are indicated as －film and strawberries with film packaging are indicated as ＋film; the temperature and humidity inside the shipment box and film packaging were recorded for －film and +film strawberries, respectively

The changes in temperature, humidity inside the shipping box, and that inside the film packaging during the storage period are shown in Figure 1. As there were no significant differences in temperature and humidity observed between the Benihoppe and Kirapika varieties during storage, only the data for Benihoppe are presented.

In the device with the temperature set to 0°C, the maximum temperature was 0.2°C, the minimum was −0.7°C, and the mean was −0.3°C both inside the shipping box and inside the film. In the device set to 3°C, the maximum temperature was 2.7°C, minimum was 2.2°C, and the mean was 2.4°C.

Humidity at 0°C storage was lower than that at 3°C storage. At both temperatures, the mean humidity was 4%–5% higher with the film packaging than without it.

The CO_2_ concentration of the air inside the film after storage at 0°C was 1.5% for Benihoppe and 1.4% for Kirapika; at 3°C, it was 1.8% for Benihoppe and 2.1% for Kirapika.

### 3.2 Decay, fresh weight, hardness, and colors

**Figure 2:**
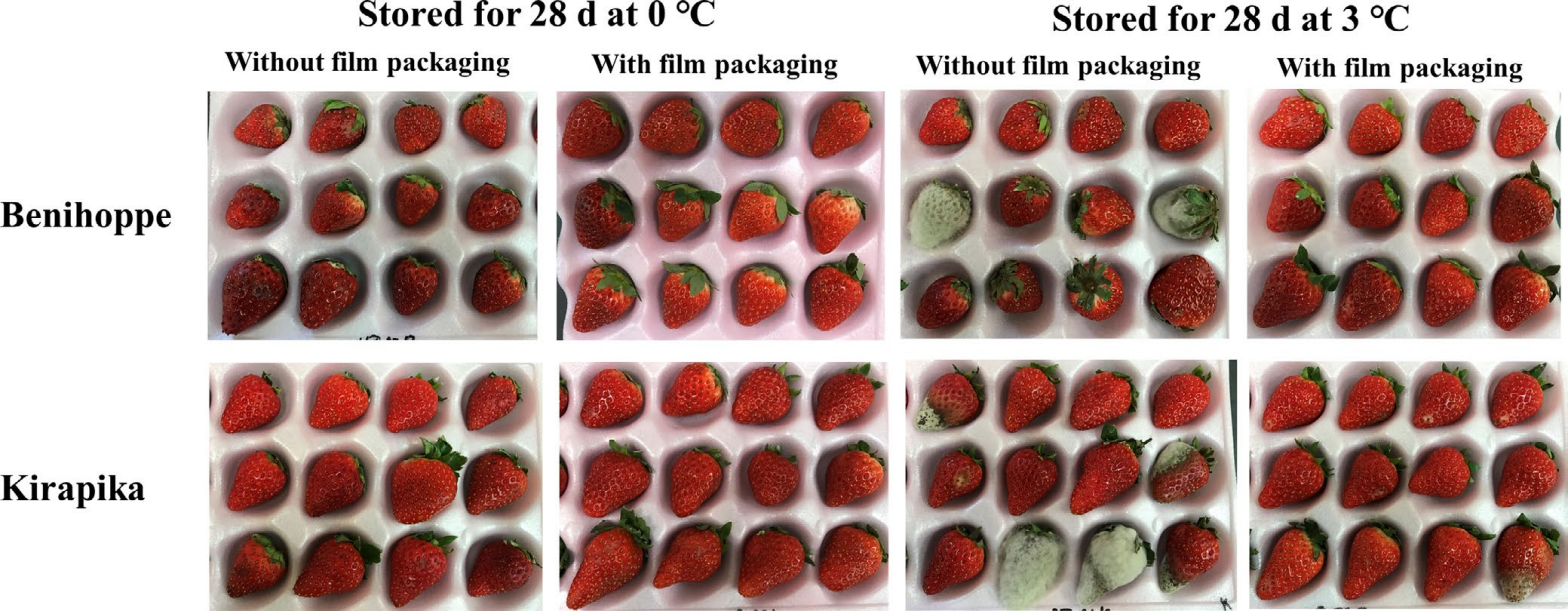
Appearance of strawberry fruits stored for 28 days

The occurrence of decay, fresh weight loss, hardness, and color after storage for 28 days is shown in Table 1, and the appearance of the strawberry fruit after storage is shown in Figure 2. For both Benihoppe and Kirapika varieties, storage at 0°C suppressed decay more effectively than that at 3°C. Furthermore, film packaging prevented the occurrence of decay in both the varieties at both storage temperatures. Notably, neither of the film‐packaged samples of the two varieties stored at 0°C for 28 days and then transferred to 20°C for 24 hr showed any decay.

**Table 1:** Difference in quality during long‐term cold storage of strawberries

Sample		Storage	Film packaging	Decay (%)	Fresh weight (%)	Hardness (*N*)	Color index		
							L^*^	a^*^	b^*^
Benihoppe	Nonstored		−	0	95.8 ± 0.2^b^	‐	28.7 ± 2.1	29.3 ± 2.2	19.8 ± 3.0
			+	0	98.8 ± 0.1^a^	2.40 ± 0.31	29.4 ± 3.0	32.0 ± 4.9	19.8 ± 3.0
	0°C	28 days at 0°C	−	10.0	88.1 ± 1.7^c^	‐	25.9 ± 3.9	26.8 ± 3.2	16.4 ± 4.4
			+	0	95.4 ± 0.3^b^	3.04 ± 0.35	31.5 ± 5.8	28.5 ± 4.5	19.4 ± 4.8
	3°C	28 days at 3°C	−	23.3	87.7 ± 1.0^c^	‐	27.3 ± 4.1	32.4 ± 4.6	17.0 ± 2.6
			+	6.6	95.6 ± 0.2^b^	2.89 ± 0.69	27.0 ± 2.6	30.2 ± 1.6	17.9 ± 3.1
Kirapika	Nonstored		−	0	95.6 ± 0.3^b^	‐	26.1 ± 1.1	31.8 ± 4.1	17.3 ± 1.7
			+	0	98.7 ± 0.1^a^	2.84 ± 0.51^a^	28.9 ± 2.3	30.6 ± 3.6	17.5 ± 3.8
	0°C	28 days at 0°C	−	16.6	86.8 ± 1.4^c^	‐	28.5 ± 5.1	27.5 ± 3.6	18.5 ± 4.8
			+	0	96.3 ± 0.4^b^	2.50 ± 0.28^a^	24.6 ± 4.4	24.5 ± 5.8	15.7 ± 6.8
	3°C	28 days at 3°C	−	53.3	85.3 ± 1.6^c^	‐	26.4 ± 5.7	31.4 ± 5.0	19.6 ± 5.0
			+	10.0	95.6 ± 0.2^b^	1.85 ± 0.27^b^	30.5 ± 1.2	29.3 ± 5.3	16.8 ± 2.7

Note

All samples were treated for 24 hr at 3°C before storage for precooling, and for 24 hr at 20°C after storage for the shelf life test. These treatments were also applied to samples not stored. Strawberries without film packaging are indicated as －film, and strawberries with film packaging are indicated as ＋film. Decay was calculated by investigating 30 fruits, and was set as decay of more than 5% of the surface area. Hardness was measured using a cylindrical plunger with a diameter of 3 mm. The fresh weight, hardness, and color index data are expressed as mean ± *SD* (*n* = 6).Superscript letters indicate different significant differences in the mean values of each variety of fresh weight, hardness, and color as assessed by Tukey's multiple range test (*p* < .05). Angle conversions were used when analyzing the fresh weight.

The results of fresh weight showed that with film packaging, the weight was maintained at 95% or more even after storage; however, without film packaging, it reduced to 90% or less. This difference was not affected by differences in storage temperature.

The hardness of strawberries stored without film packaging could not be measured using this method, because the strawberries had become semi‐dried and the cylindrical plunger did not stick during measurement; thus, break points could not be confirmed. Therefore, only data for the film‐packaged strawberries are shown. In addition, decayed strawberries were omitted from measurement.

The fruit hardness did not change even after storage at 0°C for 28 days for both Benihoppe and Kirapika. After 28 days of storage, there was a significant difference in hardness between the Kirapika samples stored at 0°C and 3°C. Upon storage at 3°C, Benihoppe showed no change in hardness compared with values before storage; whereas, Kirapika showed decreased fruit hardness.

Color did not change before or after storage for 28 days for both the varieties and at both the temperatures.

### 3.3 TSS, free sugar, and organic acid content in the juice

**Figure 3:**
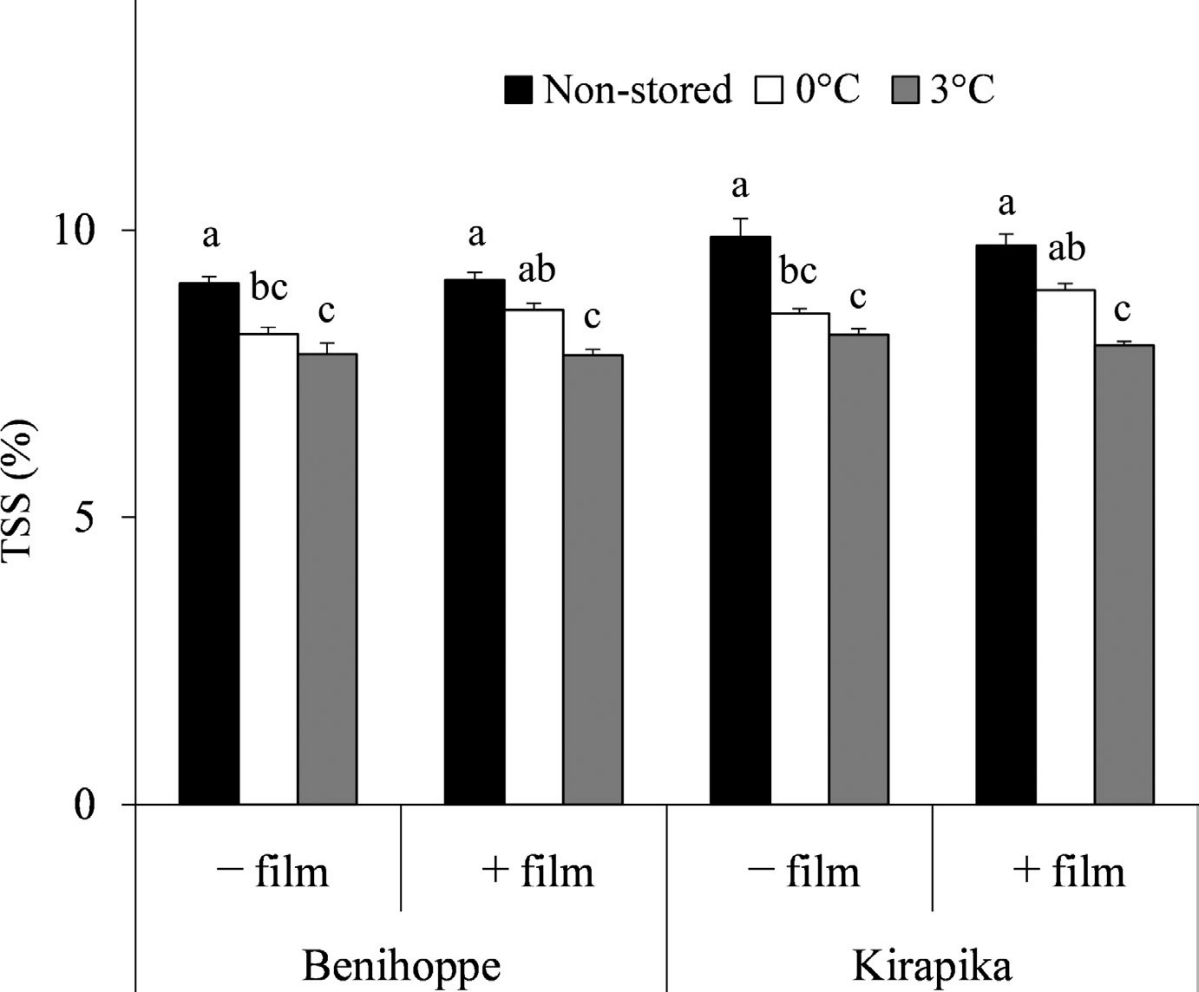
Effect of storage temperature on total soluble solid (TSS) in strawberry juice with and without film packaging. Strawberries without film packaging are indicated as －film, and strawberries with film packaging are indicated as＋film. Based on the presumption that all fruit weight reduction would be due to a reduction in water, the values of the obtained Brix were divided by the value 100(%)—weight reduction portion (%), to control for the effect of component concentration by moisture depletion. Data are expressed as mean ± *SD* (*n* = 6). Mean values of each variety with different superscript letters are significantly different by Tukey's multiple range test (*p* < .05)

The TSS contents in the juice made from strawberry fruit after storage for 28 days are shown in Figure 3. There was a significant reduction in juice TSS compared with that in nonstored juice for both the varieties when stored for 28 days under every condition except in film packaging at 0°C. Reduction in TSS was more inhibited by storage at 0°C than at 3°C for both varieties.

**Figure 4:**
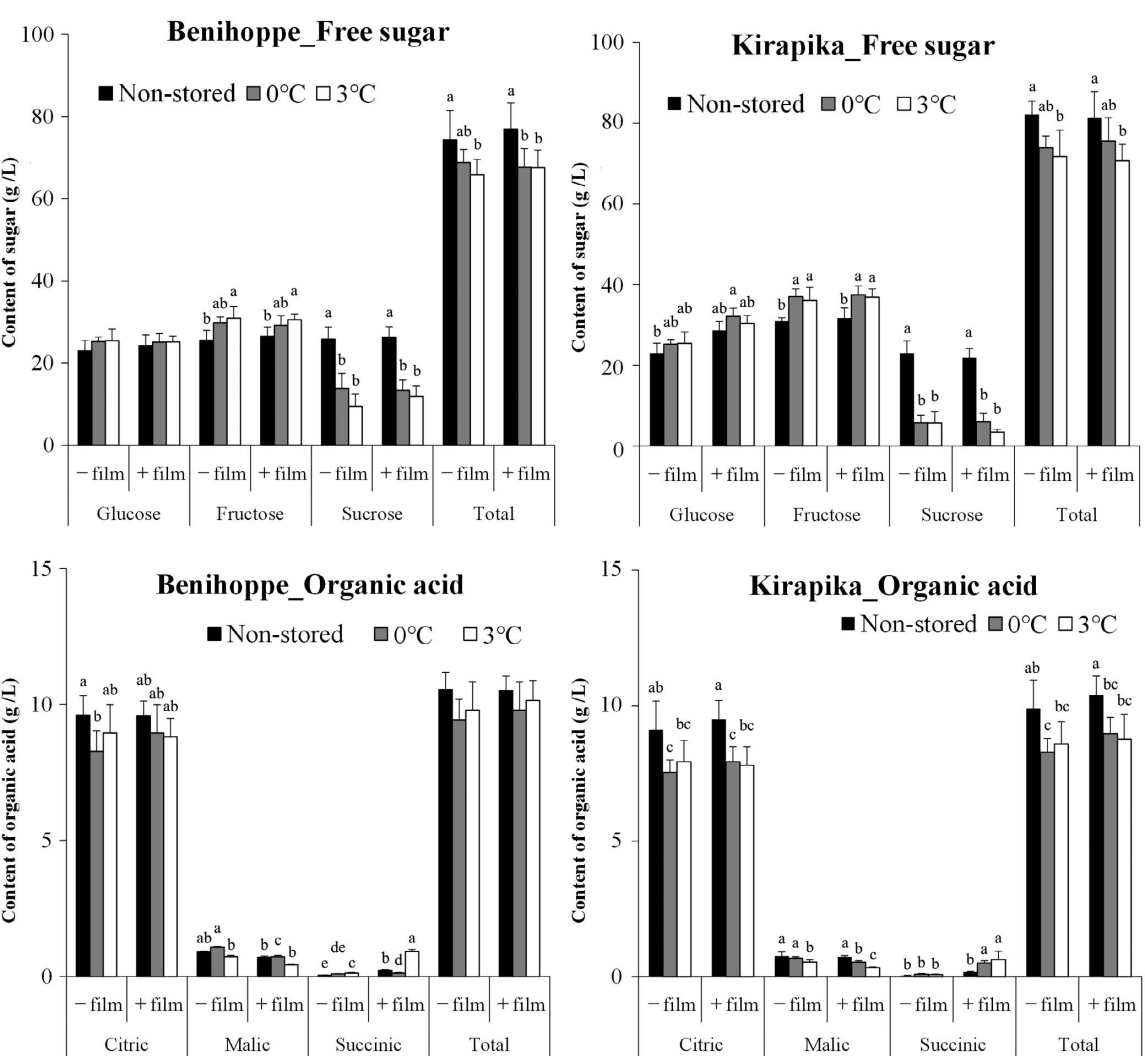
Effect of storage temperature on free sugar and organic acid in strawberry juice with and without film packaging. Strawberries without film packaging are indicated as －film, and strawberries with film packaging are indicated as＋film. Based on the presumption that all fruit weight reduction would be due to a reduction in water, the values of the obtained free sugar and organic acid are divided by the value 100(%)—weight reduction portion (%), to control for the effect of component concentration by moisture depletion. Data are expressed as mean ± *SD* (*n* = 6). Mean values of each component with different superscript letters are significantly different by Tukey's multiple range test (*p* < .05)

Free sugar and organic acid contents in the juice made from the strawberries are shown in Figure 4. There was no significant difference in glucose content before and after storage for both varieties. Fructose content was significantly increased by storage at 3°C, irrespective of the variety and use of film packaging. Fructose content was significantly increased in Kirapika at 0°C with and without film packaging, but no difference was seen in Benihoppe. On the contrary, sucrose content was significantly decreased by storage irrespective of temperature, variety, and film packaging. However, there was no difference due to storage temperature or film packaging in either variety.

For organic acids, citric acid content was significantly reduced after storage, except when Benihoppe was stored at 0°C without film packaging, and when Kirapika was stored at 3°C without film packaging. Malic acid content was lower with film packaging than without packaging in both varieties after 28 days of storage. However, succinic acid content was higher with film packaging than without packaging.

### 3.4 Evaluation of taste qualities of juices from strawberries before and after storage

The juices made from strawberries that were used in the sensory evaluations were also analyzed for TSS, free sugar, organic acid, and microbial load. The results are shown in Table 2. A comparison of the before and after values for the fruit juices showed that the TSS in Benihoppe decreased by 0.3%, and that there was no difference in Kirapika. When stored for 28 days at 0°C, there was a 3.3% reduction in fresh weight in Benihoppe and a 3.4% reduction in Kirapika. There was no decrease in TSS in Kirapika. The water content of the Kirapika strawberries was reduced during storage, which should have concentrated the juice; nevertheless, TSS has not increased.

**Table 2:** Analysis of strawberry juice used for sensory evaluation

(%)	Conditions	TSS	Free Sugar (g/L)				Organic acid (g/L)				Microbe (CFU/g)	
			Glucose	Fructose	Sucrose	Total	Citric	Malic	Succinic	Total	Aerobic	Fungi
Benihoppe	Prestorage	8.7	20.2	22.8	29.9	72.8	7.88	2.07	0.07	10.02	2.1 × 10^7^	4.5 × 10^2^
	Stored for 28 days at 0°C	8.4	22.1	26.6	23.1	71.8	7.70	1.44	0.15	9.28	1.9 × 10^7^	4.9 × 10^2^
Kirapika	Prestorage	9.3	24.3	25.7	29.8	79.8	8.07	1.62	0.08	9.78	2.0 × 10^7^	3.4 × 10^2^
	Stored for 28 days at 0°C	9.3	27.4	31.5	18.9	77.8	8.48	1.05	0.20	9.72	1.9 × 10^7^	6.0 × 10^2^

Note

Strawberries were covered with film packaging during the storage period. For sensory evaluation, the squeezed juices from many fruits were mixed and homogenized before use, these data are from the samples after mixing. The microbial tests were performed three times for each sample, and the average value was used.

Comparison for organic acid showed that citric acid was decreased in Benihoppe and increased in Kirapika. Malic acid was decreased in both the varieties. The results showed that the total amount of organic acid was decreased greatly in Benihoppe and did not change much in Kirapika.

The number of aerobic bacteria and fungi in strawberry juice did not change significantly before and after storage.

Sensory evaluation of the strawberry juices before and after storage was carried out with 40 panelists. The results are shown in Table 3. Regarding “sweetness,” there was no difference before and after storage for either variety. Regarding “sourness,” Benihoppe showed a significant decrease after storage. However, there was no difference for Kirapika during storage. There was no significant difference in the “Flavor” and “Overall taste” between the two varieties.

**Table 3:** Comparison of fruit juices of strawberries before and after storage by sensory evaluation

Attribute	Benihoppe			Kirapika		
	Prestorage	Stored		Prestorage	Stored	
Sweetness	15	25	*n*.s.	15	25	*n*.s.
Sourness	34	6	***	23	17	*n*.s.
Flavor	16	24	*n*.s.	18	22	*n*.s.
Overall preference	17	23	*n*.s.	21	19	*n*.s.

Note

"Sweetness" and "sourness" were investigated based on which felt stronger. "Flavor" and "overall preference" were investigated based on which was more preferable. (*n* = 40).

*** Indicates a significant difference according to a two‐tailed test at *p* < .001.

## 4 DISCUSSION

This study on the storage test was focused specifically on ensuring the quality of the strawberries used. We performed the test using only those fruits that had just ripened on the day of harvest. Therefore, although not many fruits were used in the test, it is considered that the results were reproducible.

The devices used for testing were set at 0°C and 3°C, with 0°C as the optimum strawberry storage temperature, and 3°C as the lowest set temperature considering an error of approximately ±2°C in the existing devices (Laguerre et al., 2002). In the device with the temperature set to 0°C, the minimum temperature was −0.7°C both inside the shipping box and inside the film. However, there were no signs of damage from freezing on the fruit. The freezing point of strawberries is reported to be around −1.1°C (Whiteman, 1977). As the temperature did not fall below this level in the storage test, it can be concluded that the strawberries did not freeze. In summary, when the device was set to 0°C or 3°C, the mean temperature tended to be 0.3–0.5°C lower than the set temperature. This appears to be a characteristic of the reefer container used for the test.

For both Benihoppe and Kirapika varieties, storage at 0°C suppressed decay more effectively than that at 3°C. No decay was observed in both the varieties with fruits in film packaging stored at 0°C. In contrast, at 3°C, some decayed fruits were seen—6.6% of Benihoppe and 10.0% of Kirapika. Fruits stored without film packaging were unsuitable for consumption due to high percentage of decay. In addition, the fresh weight of strawberries without film packaging fell to 90% or less. This finding demonstrates that simply placing strawberries in refrigeration is insufficient to inhibit reduction in fruit weight over long‐term storage; it also clarifies that the use of film packaging is required for long‐term storage. This is because the weight loss was significantly greater than 6%, which is the accepted standard for the fresh weight of strawberries for production (Nunes & Emond, 2007), even though decay occurrence was suppressed at low temperatures. In this test, the strawberries without film packaging were not of a suitable quality, even though they were not decayed. In contrast, the fruits with the film packaging were maintained at salable quality except for the decayed individuals. Therefore, storing strawberries at 0°C with film packaging is expected to be put to practical use in the future.

For fruit hardness, strawberries stored without film packaging could not be assessed because the break points were not confirmed by drying. Further their texture was unpleasant when tasted. Thus, it was considered that even if decay had not occurred, their quality was not acceptable for eating. In contrast, strawberries wrapped in film could be eaten without any problems except for the decayed ones. For strawberries wrapped in film packaging, the effect of temperature on hardness after storage differed according to the variety. Specifically, the fruit hardness of the Benihoppe variety did not show any difference between 0°C and 3°C. However, in the case of Kirapika, the hardness did not change at 0°C as compared with that before storage, but was significantly softened at 3°C. Strawberry softening during ripening mainly occurs due to the solubilization and depolymerization of cell wall components mediated by the action of enzymes (Paniagua et al., 2017; Ramos et al., 2018). Our findings thus suggest that there may be a difference in enzyme activity among different varieties.

The strawberry fruit hardens when the CO_2_ concentration is increased, even for short periods (Chandra, Choi, Lee, Lee, & Kim, 2015; Li & Kader, 1989; Nunes, Morais, Brecht, & Sargent, 2002). The treatments in these studies were conducted with CO_2_ concentrations of 5%–50%. However, as the CO_2_‐enhancing treatments have been shown to affect flavor and taste (Bovi, Caleb, Ilte, Rauh, & Mahajan, 2018), we did not introduce any additional CO_2_ in the present study, and chose a packaging film with high gas permeability, to prevent CO_2_ accumulation. The concentration of CO_2_ inside the film was less than 5% for both varieties at both storage temperatures and did not reach the CO_2_ concentrations reported in previous studies. Therefore, in this study, the gas environment created by the film packaging did not contribute to the improvement in the strawberry hardness.

When assessing the appearance of strawberries, color change is very important in addition to decay and drying. Previous research on color indicates that it does not change significantly in strawberries, even when stored at 0°C (Sacks & Shaw, 1993). This was confirmed by the results of our storage test, in which there was no significant difference in the L*, a*, b* values in relation to storage temperature or film packaging, indicating that the color did not change significantly with storage at 0°C or 3°C.

Next, we examined the changes in strawberry quality considering the TSS, sugar, and organic acid concentrations in the juice, given that strawberries with higher TSS and TSS/titratable acidity ratio are preferred (Jouquand, Chandler, Plotto, & Goodner, 2008; Rosenfeld & Ness, 2000). There was a significant reduction in juice TSS for both varieties when they were stored for 28 days under every condition except storage in film packaging at 0°C. Reduction in TSS by long‐term cold storage has also been reported in grapes (Jung, Lee, Lee, Cho, & Lee, 2018). The reduction in TSS was inhibited more by storage at 0°C than at 3°C for both the varieties. Previous research has shown that the respiration rate of strawberries is suppressed dramatically as the storage temperature decreases (Barrios, Lema, & Lareo, 2014); thus, we assume that in the present study, respiration was inhibited more at 0°C than at 3°C, which in turn inhibited the consumption of TSS, which predominantly comprises of sugar.

Furthermore, we examined the major components of TSS, which are sugars and organic acids. Both the varieties showed a greater decrease in sucrose content than an increase in fructose, demonstrating a reduction in total free sugar due to storage. Two types of sucrose metabolic pathways have been reported in fruit, involving sucrose synthase and sucrose invertase (Smith, 1999). The results of this experiment showed that fructose content increased in every kind of fruit in which sucrose content was decreased, suggesting that the sucrose had been decomposed into glucose and fructose by sucrose invertase. It is also reported that sucrose synthase activity is low in strawberries (Basson, Groenewald, Kossmann, Cronjé, & Bauer, 2010), which is also consistent with the results of the present experiment. In Benihoppe, there was no significant change in glucose content, despite the decrease in sucrose due to cold storage, indicating that the glucose had been consumed by respiration.

For organic acids, citric acid content was significantly reduced after storage. In addition, succinic acid content was higher with film packaging than without packaging. The succinic acid generation pathway has been reported as follows: succinic acid dehydrogenase activity is lost in the TCA cycle when yeast is cultured under anaerobic conditions, partially modifying the TCA cycle and generating a reduction pathway leading to succinic acid formation (Arikawa et al., 1999; Muratsubaki, 1987). The conditions inside the film are not anaerobic, but as the CO_2_ concentration is higher than that in air, it is thought that a similar reaction might have occurred partially. Malic acid is thought to be partially metabolized to succinic acid through the aforementioned reaction, resulting in a higher rate of malic acid consumption with film packaging.

In the present study, no difference in the amount of total organic acid was found in Benihoppe strawberries irrespective of storage temperature or film packaging. However, in the Kirapika variety, a significant reduction in the amount of total organic acid was observed, except when stored at 0°C without film packaging. Previous studies of varieties cultivated overseas have reported differences in organic acid metabolism during storage depending on the variety (Koyuncu & Dilmaçünal, 2010). The results of this storage test indicated the existence of varietal differences in organic acid metabolism during storage among strawberry varieties cultivated in Japan as well.

Storage test showed that storage at 0°C with film packaging was effective for the long‐term storage of strawberries. However, even under optimal conditions, long‐term storage decreases sugars and organic acids. Reduction in organic acids is said to be particularly important among the causes underlying the insipid flavor and loss of taste in stored fruit (Wang, Tanaka, Morita, & Tanaka, 1997). However, the effects of reduced sugars and organic acids on taste have not been studied.

These tests showed that strawberries could be stored for 28 days if stored at 0°C in film packaging. However, it was also clarified that the amount and composition of sugars and organic acids may change. We thus conducted additional tests to investigate the effect of these on the taste. Specifically, we compared the juice made from strawberries before and after storage at 0°C with film packaging to examine the effect of changes in the amount and composition of sugars and organic acids on taste. The reason for using juice was that strawberries before storage had to be frozen to compare with strawberries after storage and thawed frozen strawberries have a very bad texture. Thus, we have determined that using thawed strawberries is not suitable for evaluation. In this test, strawberries both before and after storage were frozen once, then thawed and squeezed for comparison. We performed microbial tests on the juices prior to sensory evaluation, to assess the risk of proliferating microorganisms during the storage of strawberries even in the absence of apparent abnormalities in appearance. We measured aerobic bacteria and fungi, as aerobic bacteria are used as an indicator of safety, and fungi are implicated as the main cause of decay in strawberry (Ayala‐Zavala et al., 2004; Sallato et al., 2007). There was barely a change in the number of these organisms after storage, compared with values before storage, indicating that aerobic bacteria and fungi were hardly increased in strawberries during the storage condition. However, these microbiological tests used frozen samples; it is expected that the results would be different with fresh strawberries.

Regarding “sweetness,” there was no difference before and after storage for either variety. This suggests that the decrease in total sugar caused by storage for 28 days at 0°C was not at a level that affected the sweetness. In addition, changes in sugar composition due to the decrease in sucrose and increases in glucose and fructose did not affect sweetness. Regarding “sourness,” Benihoppe showed a significant decrease after storage, whereas Kirapika showed no difference. Total organic acid decreased by 0.74 g/L during storage in Benihoppe, but only by 0.02 g/L in Kirapika, a difference that most likely influenced the perceived difference in sourness. The level of malic acid was greatly decreased in both varieties, and so, the ratio of citric acid was increased relatively. However, considering that there was no difference in the sourness of Kirapika, this increase in the ratio of citric acid did not seem to have significantly affected the sourness. There was no significant difference in the “Flavor,” wherein the panelists chose the juice that they most strongly preferred. In addition, there was no difference in palatability of the “Overall taste” among the two varieties. However, despite this lack of difference in palatability, there was a significant increase in the sour taste in Benihoppe after storage, suggesting that a decrease in acidity does not lower palatability. This is consistent with a previous report that showed no correlation between palatability and sourness intensity in strawberry fruits (Schwieterman et al., 2014). In summary, these findings indicate that changes in the components of strawberry juice due to storage for 28 days at 0°C does not significantly affect the consumers’ taste preferences toward the flavor of strawberries.

Overall, this study demonstrates the practicality of long‐term storage of strawberries at 0°C. Storage at 0°C reduces the occurrence of decay more than storage at 3°C does. It also suppresses softening of the fruit and reduction of sugars and organic acids, depending on the variety. Further, reduction of these sugars and organic acids does not affect strawberry palatability. However, long‐term storage of strawberries requires lowering the temperature as well as use of film packaging, which with optimal properties, prevents drying. Strawberries are difficult to store for a long duration due to the thin fruit skin and soft flesh. However, our results suggest that strawberries can be stored for more than 28 days using appropriate devices and packaging materials.

## CONFLICT OF INTEREST

The authors declare no conflict of interest or relationship, financial or otherwise.

## ETHICAL APPROVAL

This study was approved by the Institutional Review Board of Shizuoka Prefectural Research Institute of Agriculture and Forestry.

## INFORMED CONSENT

Informed consent was obtained from all individual participants included in the study.

## References

[fsn31817-bib-0001] Angelini R. (Ed.) (2010). La fregola (in Italian). Milan: Bayer Crop Science.

[fsn31817-bib-0002] Arikawa, Y. , Kuroyanagi, T. , Shimosaka, M. , Muratsubaki, H. , Enomoto, K. , Kodaira, R. , & Okazaki, M. (1999). Effect of gene disruptions of the TCA cycle on production of succinic acid in *Saccharomyces cerevisiae* . Journal of Bioscience and Bioengineering, 87, 28–36. 10.1016/S1389-1723(99)80004-8 16232421

[fsn31817-bib-0003] Ayala‐Zavala, J. F. , Wang, S. Y. , Wang, C. Y. , & González‐Aguilar, G. A. (2004). Effect of storage temperatures on antioxidant capacity and aroma compounds in strawberry fruit. LWT ‐ Food Science and Technology, 37, 687–695. 10.1016/j.lwt.2004.03.002

[fsn31817-bib-0004] Bang, J. , Lim, S. , Yi, G. , Lee, J. G. , & Lee, E. J. (2019). Integrated transcriptomic‐metabolomic analysis reveals cellular responses of harvested strawberry fruit subjected to short‐term exposure to high levels of carbon dioxide. Postharvest Biology and Technology, 148, 120–131. 10.1016/j.postharvbio.2018.11.003

[fsn31817-bib-0005] Barrios, S. , Lema, P. , & Lareo, C. (2014). Modeling respiration rate of strawberry (cv. San Andreas) for modified atmosphere packaging design. International Journal of Food Properties, 17, 2039–2051. 10.1080/10942912.2013.784328

[fsn31817-bib-0006] Basson, C. E. , Groenewald, J.‐H. , Kossmann, J. , Cronjé, C. , & Bauer, R. (2010). Sugar and acid‐related quality attributes and enzyme activities in strawberry fruits: Invertase is the main sucrose hydrolysing enzyme. Food Chemistry, 121, 1156–1162. 10.1016/j.foodchem.2010.01.064

[fsn31817-bib-0007] Bovi, G. G. , Caleb, O. J. , Ilte, K. , Rauh, C. , & Mahajan, P. V. (2018). Impact of modified atmosphere and humidity packaging on the quality, off‐odour development and volatiles of ‘Elsanta’ strawberries. Food Packaging and Shelf Life, 16, 204–210. 10.1016/j.fpsl.2018.04.002

[fsn31817-bib-0008] Chandra, D. , Choi, A. , Lee, J. S. , Lee, J. , & Kim, J. (2015). Changes in physicochemical and sensory qualities of “Goha” strawberries treated with different conditions of carbon dioxide. Agricultural Sciences, 6, 325–334. 10.4236/as.2015.63033

[fsn31817-bib-0009] Eaks, I. L. , & Morris, L. L. (1956). Respiration of cucumber fruits associated with physiological injury at chilling temperatures. Plant Physiology, 31, 308–314. 10.1104/pp.31.4.308 16654887 PMC540788

[fsn31817-bib-0010] Filho, M. J. , Scolforo, C. Z. , Saraiva, S. H. , Pinheiro, C. J. G. , Silva, P. I. , & Lucia, S. M. D. (2018). Physicochemical, microbiological and sensory acceptance alterations of strawberries caused by gamma radiation and storage time. Scientia Horticulturae, 238, 187–194. 10.1016/j.scienta.2018.04.053

[fsn31817-bib-0011] Jouquand, C. , Chandler, C. , Plotto, A. , & Goodner, K. (2008). A sensory and chemical analysis of fresh strawberries over harvest dates and seasons reveals factors that affect eating quality. Journal of the American Society for Horticultural Science, 133, 859–867. 10.21273/JASHS.133.6.859

[fsn31817-bib-0012] Jung, H. M. , Lee, S. , Lee, W. , Cho, B. , & Lee, S. (2018). Effect of vibration stress on quality of packaged grapes during transportation. Engineering in Agriculture, Environment and Food, 11, 79–83. 10.1016/j.eaef.2018.02.007

[fsn31817-bib-0013] Kawata, T. , Takeuchi, T. , Ikari, T. , Mochizuki, M. , Oishi, T. , Saiki, C. , … Goto, Y. (2016). Fruit characteristics of the new strawberry cultivar ‘Kirapika’. Bulletin Shizuoka Research Institute of Agriculture and Forestry, 9, 1–10. (in Japanese).

[fsn31817-bib-0014] Koyuncu, M. A. , & Dilmaçünal, T. (2010). Determination of vitamin C and organic acid changes in strawberry by HPLC during cold storage. Notulae Botanicae Horti Agrobotanici Cluj‐Napoca, 38, 95–98.

[fsn31817-bib-0015] Ktenioudaki, A. , O’Donnell, C. P. , & Nunes, M. C. N. (2019). Modelling the biochemical and sensory changes of strawberries during storage under diverse relative humidity conditions. Postharvest Biology and Technology, 154, 148–158. 10.1016/j.postharvbio.2019.04.023

[fsn31817-bib-0016] Laguerre, O. , Derens, E. , & Palagos, B. (2002). Study of domestic refrigerator temperature and analysis of factors affecting temperature: A French survey. International Journal of Refrigeration, 25, 653–659. 10.1016/S0140-7007(01)00047-0

[fsn31817-bib-0017] Li, C. , & Kader, A. A. (1989). Residual effects of controlled atmospheres on postharvest physiology and quality of strawberries. Journal of the American Society for Horticultural Science, 114, 629–634.

[fsn31817-bib-0018] Matsumoto, K. , Lee, C. , Chun, J. , Kim, T. , Tamura, F. , Tanabe, K. , & Hwang, Y. (2008). Varietal differences of fruit quality and shelf life in strawberry cultivars developed in Korea. Horticultural Research (Japan), 7, 293–297. (In Japanese with English abstract). 10.2503/hrj.7.293

[fsn31817-bib-0019] Meilgaard M., Civille G., & Carr T. (Eds.) (2007). Sensory evaluation technique, 4th ed. New York, NY: CRC Press.

[fsn31817-bib-0020] Mochizuki, M. , Kawata, T. , Ikegaya, A. , Ikari, T. , Goto, Y. , & Takeuchi, T. (2018). Fruit characteristics of the new strawberry cultivar “Kirapika”. Bulletin Shizuoka Research Institute of Agriculture and Forestry, 11, 49–54. (in Japanese).

[fsn31817-bib-0021] Morris, G. J. , & Clarke, A. (1981). Effect of low temperatures on biological membranes. London: Academic Press.

[fsn31817-bib-0022] Muratsubaki, H. (1987). Regulation of reductive production of succinate under anaerobic conditions in baker’s yeast. Journal of Biochemistry, 102, 705–714. 10.1093/oxfordjournals.jbchem.a122108 3325498

[fsn31817-bib-0023] Nunes, C. N. , & Emond, J. P. (2007). Relationship between weight loss and visual quality of fruits and vegetables. Proceedings of the Florida State Horticultural Society, 120, 235–245.

[fsn31817-bib-0024] Nunes, C. N. , Morais, A. M. M. B. , Brecht, J. K. , & Sargent, S. A. (2002). Fruit maturity and storage temperature influence response of strawberries to controlled atmospheres. Journal of the American Society for Horticultural Science, 127, 836–842. 10.21273/JASHS.127.5.836

[fsn31817-bib-0025] Ozkaya, O. , Dündar, O. , Scovazzo, G. C. , & Volpe, G. (2009). Evaluation of quality parameters of strawberry fruits in modified atmosphere packaging during storage. African Journal of Biotechnology, 8, 789–793.

[fsn31817-bib-0026] Paniagua, C. , Santiago‐Doménech, N. , Kirby, A. R. , Gunning, A. P. , Morris, V. J. , Quesada, M. A. , … Mercado, J. A. (2017). Structural changes in cell wall pectins during strawberry fruit development. Plant Physiology and Biochemistry, 118, 55–63. 10.1016/j.plaphy.2017.06.001 28618373

[fsn31817-bib-0027] Paparozzi, E. T. , Meyer, G. E. , Schlegel, V. , Blankenship, E. E. , Adams, S. A. , Conley, M. E. , … Reade, P. E. (2018). Strawberry cultivars vary in productivity, sugars and phytonutrient content when grown in a greenhouse during the winter. Scientia Horticulturae, 227, 1–9. 10.1016/j.scienta.2017.07.048

[fsn31817-bib-0028] Park, J. E. , Kim, H. M. , & Hwang, S. J. (2012). Effect of harvest time, precooling, and storage temperature for keeping the freshness of “Maehyang” strawberry for export. Journal of Bio‐Environment Control, 21, 404–410. 10.12791/KSBEC.2012.21.4.404

[fsn31817-bib-0029] Pott, D. M. , Lima, F. A. , Soria, C. , Willmitzer, L. , Fernie, A. R. , Nikoloski, Z. , … Vallarino, J. G. (2020). Metabolic reconfiguration of strawberry physiology in response to postharvest practices. Food Chemistry, 321, 126747 10.1016/j.foodchem.2020.126747 32276147

[fsn31817-bib-0030] Ramos, P. , Parra‐Palma, C. , Figueroa, C. , Zuñiga, P. , Valenzuela‐Riffo, F. , Gonzalez, J. , … Morales‐Quintana, L. (2018). Cell wall‐related enzymatic activities and transcriptional profiles in four strawberry (*Fragaria × ananassa*) cultivars during fruit development and ripening. Scientia Horticulturae, 238, 325–332.

[fsn31817-bib-0031] Rodríguez‐Bermejo, J. , Barreiro, P. , Robla, J. I. , & Ruiz‐García, L. (2007). Thermal study of a transport container. Journal of Food Engineering, 80, 517–527. 10.1016/j.jfoodeng.2006.06.010

[fsn31817-bib-0032] Rosenfeld, H. , & Ness, A. (2000). Prediction of sensory quality of strawberry jam by means of sensory quality attributes of fresh fruits. Journal of the Science of Food and Agriculture, 80, 1895–1902. 10.1002/1097-0010(200010)80:13<1895:AID-JSFA717>3.0.CO;2-C

[fsn31817-bib-0033] Sacks, E. J. , & Shaw, D. V. (1993). Color change in fresh strawberry fruit of seven genotypes stored at 0°C. HortScience, 28, 209–210. 10.21273/HORTSCI.28.3.209

[fsn31817-bib-0034] Sallato, B. V. , Torres, R. , Zoffoli, J. P. , & Latorre, B. A. (2007). Effect of boscalid on postharvest decay of strawberry caused by *Botrytis cinerea* and *Rhizopus stolonifera* . Spanish Journal of Agricultural Research, 5, 67–78. 10.5424/sjar/2007051-224

[fsn31817-bib-0035] Schwieterman, M. L. , Colquhoun, T. A. , Jaworski, E. A. , Bartoshuk, L. M. , Gilbert, J. L. , Tieman, D. M. , … Clark, D. G. (2014). Strawberry flavor: Diverse chemical compositions, a seasonal influence, and effects on sensory perception. PLoS One, 9, e88446 10.1371/journal.pone.0088446 24523895 PMC3921181

[fsn31817-bib-0036] Smith, H. B. (1999). Sucrose synthase and the fruit of its labor. The Plant Cell, 11, 2261–2262. 10.1105/tpc.11.12.2261 10590155 PMC526002

[fsn31817-bib-0037] Takeuchi, T. , Fujinami, H. , Kawata, T. , & Matsumura, M. (1999). Pedigree and characteristics of a new strawberry cultivar ‘Benihoppe’. Bulletin Shizuoka Agricultural Experiment Station, 44, 13–24. (in Japanese).

[fsn31817-bib-0038] Wang, S. , Tanaka, S. , Morita, K. , & Tanaka, F. (1997). Basic studies on the storage method of strawberry. Nogyo Shisetsu, 27, 207–215. (in Japanese).

[fsn31817-bib-0039] Whiteman, T. M. (1977). Freezing point of fruits and vegetables. Washington, D.C: Department of Agriculture, Agricultural Research Service.

[fsn31817-bib-0040] Yan, J. , Luo, Z. , Ban, Z. , Lu, H. , Li, D. , Yang, D. , … Li, L. (2019). The effect of the layer‐by‐layer (LBL) edible coating on strawberry quality and metabolites during storage. Postharvest Biology and Technology, 147, 29–38. 10.1016/j.postharvbio.2018.09.002

